# Complement 3a Mediates CCN2/CTGF in Human Retinal Pigment Epithelial Cells

**DOI:** 10.1155/2022/3259453

**Published:** 2022-07-30

**Authors:** Kang Xiao, Zhiyan Xu, Zhengyu Chen, Qin Long, Youxin Chen

**Affiliations:** ^1^Department of Ophthalmology, Peking Union Medical College Hospital, Chinese Academy of Medical Sciences & Peking Union Medical College, Beijing, China; ^2^Key Laboratory of Ocular Fundus Diseases, Chinese Academy of Medical Sciences, Beijing, China

## Abstract

**Background:**

Complement 3 (C3) is the crucial component of the complement cascade when retina was exposed to external stimulus. Cellular communication network 2/connective tissue growth factor (CCN2/CTGF) is important in response of retinal stress and a fulcrum for angiogenesis and fibrosis scar formation. Our study aims to explore the interaction between C3 and CCN2/CTGF via bioinformatics analyses and *in vitro* cell experiments.

**Methods:**

The GSE dataset was selected to analyse the chemokine expression in human retinal pigment epithelium (ARPE-19) cells under stimulus. Then, RPE cells were further transfected with or without C3 siRNA, followed by C3a (0.1 *μ*M or 0.3 *μ*M) for 24, 48, and 72 hours. Reverse transcription-polymerase chain reaction (RT-PCR) and enzyme-linked immunosorbent assay (ELISA) were used to measure CCN2/CTGF mRNA and protein levels.

**Results:**

The GSE36331 revealed C3 expression was significantly elevated in RPE under stimulus. Compared with negative control, CCN2/CTGF mRNA was increased with all types of C3a treatments, whereas a significant increase of protein level was only observed with high concentration of 0.3 *μ*M C3a for a prolonged 72-hour time. Compared with nontransfected cells, significant reductions of CCN2/CTGF mRNA were observed in the C3 siRNA transfected cells with 0.3 *μ*M C3a for 24, 48, and 72 hours, and a significant reduction of CCN2/CTGF protein was observed with 0.3 *μ*M C3a for 48 hours.

**Conclusions:**

C3 was elevated in RPE under environmental stimulus and long-term exposure to specified concentration of C3a increased CCN2/CTGF expression in RPE, which could be partially reversed by C3 siRNA.

## 1. Introduction

Several studies have demonstrated the harmfulness of pathological vascular response under stimulus on retinal health and function, which causes haemorrhage, exudation, retinal pigment epithelial detachment, disciform fibrosis scar, and vision loss [1, 2]. These retinal vasculopathies are multifactorial and caused by the combination of hypoxic stress, inflammatory response, and endothelial and extracellular matrix (ECM) dysfunction [3]. ECM acts as an essential support, regulating the scaffold of retinal development and vascular pathologies [4]. One major profibrotic factor that promotes excessive ECM accumulation is cellular communication network 2 (CCN2), also known as connective tissue growth factor (CTGF), which is essential for vascular development and the modulation of cell movement [5].

CCN2/CTGF is a highly conserved gene among vertebrate species. It is overexpressed together with increased ECM synthesis in fibrovascular diseases in many organs, such as kidney fibrosis [6] and idiopathic pulmonary fibrosis [7]. Studies showed that CCN2/CTGF and transforming growth factor (TGF)-*β* acted in a cooperative manner to promote fibrosis [8]. It was reported that CCN2/CTGF enhanced TGF-*β*-induced mesangial cell phosphorylation, Smad2, and Smad3 nuclear translocation [9]. Moreover, CCN2/CTGF could activate numerous signalling molecules, such as ERK1/2, JNK and PKB via one or more signalling receptors, which played a role in the fibrotic disease [10]. CCN2/CTGF is also expressed in the vascular endothelial cells around the retina [11], mural cells, and retinal pigment epithelial (RPE) cells [12]. The functions of CCN2/CTGF in many ocular diseases have been clarified. In diabetic retinopathy (DR), the hallmark pathological changes are basal lamina (BL) thickening of the retinal capillary, which is caused by increased advanced glycation end products (AGEs) via the TGF-*β*-CTGF pathways [13, 14]. The accumulation of CCN2/CTGF is also found in the contractile membranes of proliferative vitreoretinopathy (PVR) patients and in subretinal fluid during retinal detachment [15]. High levels of CCN2/CTGF are also involved in choroidal neovascularisation (CNV). *In vivo,* mice CNV model studies prove that the inhibition of CCN2/CTGF induces a significant reduction in lesion size [16, 17]. Overall, with the ECM components, CCN2/CTGF plays a pivotal role in responding to retinal stress and serves as a fulcrum for angiogenesis and fibrotic scar formation.

The retina has a unique immune adjusting system and the innate immune system plays a key role in responding to external stimuli, among which the most classical component is the complement cascade. There are 3 pathways (classical, lectin, and alternative) to initiate the complement cascade by converting complement 3 (C3), the key component in the complement cascade, to C3a, which marks the activation of the complement system, with the final product formation of the membrane attack complex (MAC) and cell lysis [18,19]. Recently, the role of C3 on the retinal fibrovascular diseases has received considerable attention. Increased plasma C3a protein was found in age-related macular degeneration (AMD) patients, compared with control [20]. It was also found that mice injected with C3-expressing adenovirus exhibited significantly increased vascular permeability, endothelial cell proliferation and migration, RPE atrophy, and reduced retinal function relative to those injected with a control adenovirus, which reproduce many of the features of AMD and other retinal diseases, such as DR and proliferative vitreoretinopathy [21].

Most retinal fibrovascular diseases are wound healing-like responses in which neovascularisation is often accompanied by influx of inflammatory cells and myofibroblasts into the retina causing fibrosis of the retina [14, 22]. Nowadays, angiogenesis is considered to play a major role in the retinal fibrovascular diseases, and the anti-VEGF therapy has been widely used to inhibit angiogenesis. However, more studies indicate that enhanced or persistent fibrotic responses are also the main factor preventing substantial visual improvement and pursue the therapeutic option for regulating angio-fibrotic switch and decreasing fibrosis [23]. Recently, experimental evidences suggested that CCN2/CTGF played a role of in the angio-fibrotic switch and was a causal factor of fibrosis and scarring in retinal fibrovascular diseases [24, 25]. Hence, exploring strategy targeting CCN2/CTGF is of great significance, which may provide a novel sight in treating retinal fibrovascular diseases.

In a study about tubulointerstitial renal fibrosis, C3a treatment increased the mRNA and protein levels of CCN2/CTGF in human proximal tubular epithelial cell line (HK-2) [26]. Moreover, specific inhibition of complement with compstatin (a C3 inhibitor) downregulated mRNA and protein expressions of CCN2/CTGF in lung tissue of sepsis-induced fibrosis model [27]. Hence, C3 may regulate the expression of CCN2/CTGF in the fibroproliferative response. However, the interaction between them remains unclarified in retinal fibrovascular diseases. Thus, our study aimed to uncover the relationships between C3 and CCN2/CTGF in RPE cells. We first investigated previous microarray datasets and performed *in silico* analyses to identify molecular changes in RPE under an environmental stimulus. Then, an *in vitro* RPE cell line model was applied to validate the bioinformatics results.

## 2. Materials and Methods

### 2.1. Microarray Analysis

Microarray datasets of chemokine expression in human RPE cell lines (ARPE-19) in response to co-culture with activated T cells (GSE36331, https://www.ncbi.nlm.nih.gov/geo/query/acc.cgi?acc=GSE36331; platform GPL6244, https://www.ncbi.nlm.nih.gov/geo/query/acc.cgi?acc=GPL6244) were analyzed using *R* (v3.6.3) with Bioconductor packages (https://www.bioconductor.org/). The probes were annotated using package “hugene10sttranscriptcluster.db.” Limma was used to obtain differential gene expression. Molecular Signatures Database (MSigDB) v7.4 was used for the gene set enrichment analysis (GSEA) (https://www.gsea-msigdb.org/gsea/msigdb/). The resulting false discovery rates (FDR) were corrected for multiple testing, genes with adjusted *P*-value < 0.01 were marked as significant, logFC >1 were upregulated genes, logFC < −1 were downregulated genes.

### 2.2. Cell Cultures and Treatment

The human ARPE-19 cell line was obtained from the Eye Laboratory of Peking University People's Hospital. The cells were cultured in DMEM-F12 (Gibco from ThermoFisher, Carlsbad, CA, USA) containing 100U/ml streptomycin/penicillin and 15% fetal bovine serum (FBS) at 37°C in a humidified environment containing 5% CO2. The cells were passaged twice weekly to maintain cell confluence around 70%.

A total of 4 × 10^5^ cells were seeded into six-well plates with a fresh medium for 48 hours, followed by starvation in serum-free MEM for 24 hours. Then, the cells were treated with 0.1 *μ*M or 0.3 *μ*M recombinant human complement C3a (Sigma-Aldrich, Cat#204881) for 24, 48, and 72 hours. Negative controls were cells with no short interfering RNA (siRNA) transfection or C3a treatment.

### 2.3. C3 Gene Silencing Using siRNA

Three duplex siRNAs (Origene, Cat#SR300499) were applied to target human *C3* (NCBI: NM_000064.4). Meanwhile, a nonsilencing siRNA duplex targeting sequence, AATTCTCCGAACGTGTCACGT, was used as a scramble control. The cells were seeded in six-well plates at a density of 5 × 10^5^ cells/well until 70–80% confluence was reached. siRNA transfection was performed with Lipofectamine® RNAiMAX (Life Technologies) in culture medium (without FBS and antibiotics) according to the manufacturer's instructions. Briefly, 30pmol of siRNA was diluted with 150 *μ*l of Opti-MEM medium, and this was mixed with a diluted transfection reagent (9 *μ*l of transfection reagent in 141 *μ*l Opti-MEM medium). After incubating at room temperature for 5 minutes, the siRNA-lipid complex was added to the cells. The transfected cells were incubated for another 48 hours. Then, a reverse transcription-polymerase chain reaction (RT-PCR) analysis was performed to examine the gene silencing effect.

### 2.4. RNA Isolation and Semiquantitative RT-PCR

Total RNA was isolated from the cultured RPE cells using the TRIZOL-chloroform method. Aliquots of total RNA were reverse-transcribed into single-stranded cDNA using Moloney Murine Leukemia Virus-derived reverse transcriptase (MMLV RT) (Takara Bio, Cat# 6110A). A fixed PCR cycle was performed on a GeneAmp 9700 system using diluted cDNA product, *Ex Taq* DNA polymerase (Takara Bio, RR006 A), and primers specific for C3a, GAPDH, and CCN2/CTGF (listed in Table 1). GAPDH RNA was used as the internal control. The semiquantitative analysis was achieved by comparing the PCR products normalised to the internal control. The PCR products were visualised on agarose gels with ethidium bromide and analysed using ImageJ (National Institutes of Health).

### 2.5. Enzyme-Linked Immunosorbent Assay (ELISA)

The conditioned media were collected after C3a and siRNA treatment as described above. Concentrations of CCN2/CTGF (Biofine, Inc., Beijing, China) were determined with an ELISA kit according to the manufacturer's instructions. The results were normalised to the negative control.

### 2.6. Statistical Analysis

Data were analyzed using IBM SPSS 23.0 (SPSS, Chicago, IL) and GraphPad Prism 8 (GraphPad Software, La Jolla, CA) was used to generate figures. All data are expressed as mean ± standard deviation (SD). A one-way analysis of variance (ANOVA) with Tukey's post comparison test was used for multiple comparisons. Differences between two groups were evaluated using nonpaired Student's *t*-test. *P* < 0.05 was considered statistically significant.

## 3. Results

### 3.1. RPE Cells Cocultured with Activated T-cells Show an Increased Inflammatory and Complement Response via a Bioinformatics Analysis

**Figure 1:**
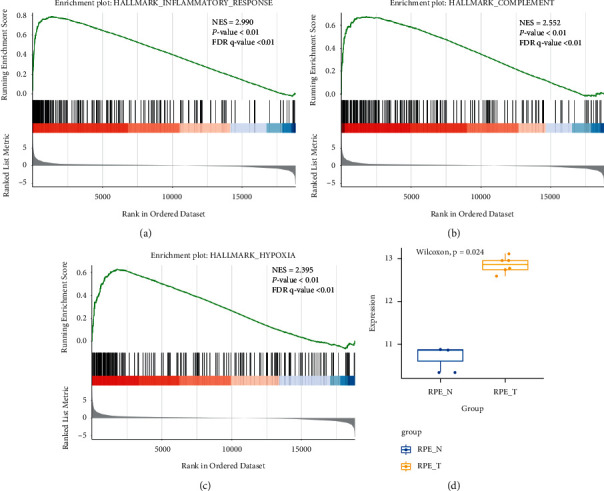
Bioinformatics re-analysis of dataset GSE36331. In RPE cells cocultured with activated T-cells, gene sets for inflammatory response, complement, and hypoxia were significantly enriched in the RPE_T group compared to the RPE_N group (a-c). The normalised enrichment score (NES) is used to compare the enrichment results across the gene sets. The *y*-axis is the enrichment score (ES), and the *x*-axis represents the genes (parallel vertical black lines) included in the gene sets. The green line is composed of each ES from the genes. The ES calculated for each gene represents the degree of over-expression at the top left or the bottom right along with the ranked gene list. The red colour band below represents a positive correlation for an enriched pathway. On the contrary, the blue colour is for the negative correlation. FDR q-values < 0.05 were considered as significant thresholds. The box plot illustrates the differential expression level of C3 between the RPE_N and RPE_T groups (d). Nonparametric Wilcoxon test *P* values are shown on top of the graph, and the boxes represent the median and quartiles of the gene expression values.

The GSE36331 dataset was extracted from the Gene Expression Omnibus (GEO). RPE cells cultured alone (RPE_N, *n* = 3) and RPE cells cocultured with activated T-cells (RPE_T, *n* = 6 in total, 2 with T-cells alone, 2 with additional anti-TNF*α*, 2 with additional anti-INF*γ*) were included for the differential gene expression. The gene expression data were extracted, and GSEA was performed after the differential gene expression analysis. The hallmark gene sets were used for the enrichment of specific predefined biological processes. Inflammatory response, complement, and hypoxia were among the top enriched gene sets (Figures 1(a)–1(c), FDR q-value<0.01). The expression of major C3 was significantly higher in RPE_T than in the RPE_N group (Figure 1(d), ^*∗*^*P*=0.024).

### 3.2. Expression of CNN2/CTGF Is Elevated in RPE Cells upon Long-Term Exposure to C3a

**Figure 2:**
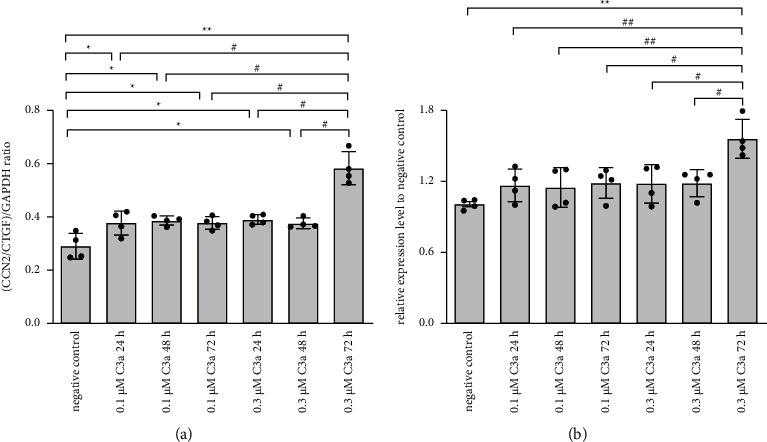
Effect of C3a on mRNA and protein levels of CCN2/CTGF in cultured RPE cells. Total RNA was extracted, and CCN2/CTGF mRNA was evaluated by RT-PCR analysis. The ratio of the abundance of the target mRNA to GAPDH was evaluated by densitometric analysis (a). ^*∗*^*P* < 0.05, ^*∗∗*^*P* < 0.01versus negative control cells; #*P* < 0.05, ##*P* < 0.01 versus cells with 0.3 *μ*M C3a for 72-hour time. ELISA analysis to assess the effect of C3a on protein levels of CCN2/CTGF in cultured RPE cells (b). The expression level was normalised to the negative control. ^*∗*^*P* < 0.05, ^*∗∗*^*P* < 0.01 versus negative control; #*P* < 0.05, ##*P* < 0.01 versus cells with 0.3 *μ*M C3a for 72-hour time. The data are presented as the mean ± SD (*n* = 4).

To detect whether the expression of CCN2/CTGF was related to C3a, RPE cells were treated with either 0.1 *μ*M or 0.3 *μ*M C3a for 24, 48, and 72 hours. Semiquantitative RT-PCR and ELISA were performed to compare the relative CCN2/CTGF mRNA and protein levels under the different conditions (Figure 2). The CCN2/CTGF mRNA level significantly increased with all types of C3a treatments, and the degree of increase was higher with 0.3 *μ*M C3a for 72 hours than that with lower dosage and less time of C3a (Figure 2(a)). The CCN2/CTGF protein level increased significantly when cells were treated with 0.3 *μ*M C3a for 72 hours, and the lower dosage and less time of C3a showed a tendency to increase, but this trend was statistically insignificant (Figure 2(b)).

### 3.3. Effects of siRNA Targeting C3 on the mRNA and Protein Levels of CCN2/CTGF in Cultured RPE Cells Treated with Exogenous C3a

To detect if the siRNA targeting C3 could reverse the elevated CCN2/CTGF mRNA and protein expressions in RPE cells with exogenous C3a treatment, three sets of duplex siRNAs (SR300499A, B, C) targeting C3a were applied for gene silencing. RPE cells showed efficient gene-silencing of C3a using siRNA transfection. C3a mRNA was not detected in SR300499A and SR300499B, whilst the expression of the internal control of GAPDH was the same. siRNA SR300499A was used in the following experiment, which was the same as our previous study (results not shown here) [28].

**Figure 3:**
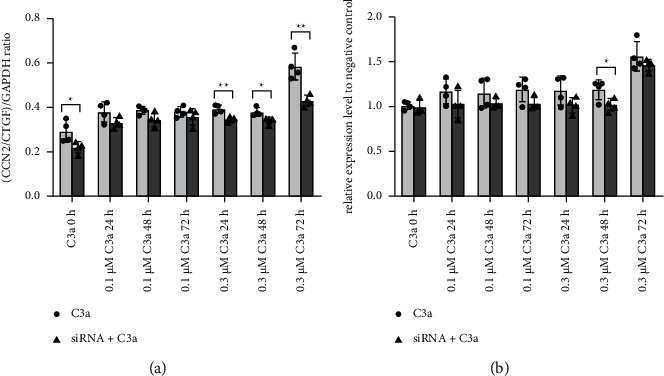
Effects of C3 siRNA on the mRNA and protein levels of CCN2/CTGF in RPE cells with exogenous C3a treatment. Total RNA was extracted, and CCN2/CTGF mRNA was evaluated by RT-PCR analysis. The ratio of the abundance of the target mRNA to GAPDH was evaluated by a densitometric analysis (a) ^*∗*^*P* < 0.05, ^*∗∗*^*P* < 0.01 versus respective non-transfected cells. ELISA analysis of the effect of C3 siRNA on the protein level of CCN2/CTGF in RPE cells with exogenous C3a treatment (b). The expression level was normalised to the negative control. ^*∗*^*P* < 0.05, ^*∗∗*^*P* < 0.01 versus respective nontransfected cells. The data are presented as the mean ± SD (*n* = 4).

RPE cells were transfected by siRNA to silence C3a expression transiently. Forty-eight hours after transfection, the cells were treated with different doses of human recombinant C3a for various periods (Figure 3). Compared to nontransfected cells, CCN2/CTGF mRNA level decreased when the cells were transfected with siRNA, and the reduction was significant in the transfected cells treated with 0.3 *μ*M C3a for 24, 48, and 72 hours (Figure 3(a)). Moreover, the CCN2/CTGF protein level in transfected cells did not decrease significantly compared to nontransfected cells in most groups. A significant reduction of CCN2/CTGF protein was only observed in the transfected cells treated with 0.3 *μ*M C3a for 48 hours (Figure 3(a)).

## 4. Discussion

**Figure 4:**
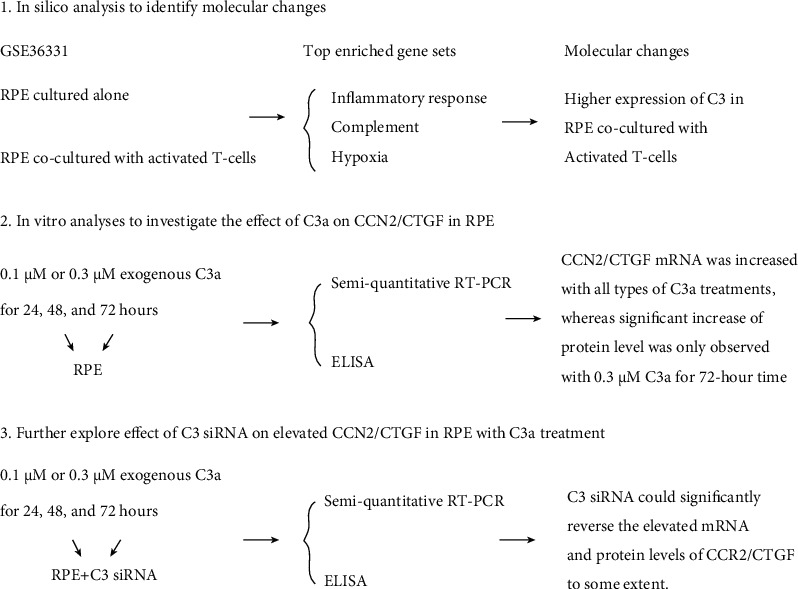
Summary of relationship between C3 and CCN2/CTGF via bioinformatics analyses and *in vitro* cell experiments.

Our study demonstrated that C3 was elevated in RPE under an environmental stimulus via bioinformatics analyses. Moreover, we explore the interaction between C3 and CCN2/CTGF in cell experiments. With exogenous C3a treatment, the mRNA and protein expressions of CCN2/CTGF in RPE was increased to some extent. We further conducted C3 siRNA transfection in RPE with exogenous C3a treatment. Compared with non-transfected cells, siRNA targeting C3 could partly reverse the elevated CCN2/CTGF mRNA and protein levels in RPE with exogenous C3a treatment (Figure 4).

In the bioinformatic analysis section, the GSE36331 dataset was re-analysed. Human RPE under environmental stimulus showed enrichment of gene sets for inflammatory response, complement, and hypoxia. The essential compartment component in the complement cascade, C3, showed a significantly higher expression in stimulated RPE versus control. We further investigated the relationship between C3 and CCN2/CTGF in RPE cells *in vitro*. Our results revealed that CCN2/CTGF mRNA levels were slightly increased with 0.1 *μ*M C3a (24, 48, and 72 hours) and 0.3 *μ*M C3a (24 and 48 hours), whilst the corresponding changes of protein level were insignificant, indicating the effect of lower concentration or shorter time of C3a on CCN2/CTGF expression was limited. Moreover, CCN2/CTGF mRNA and protein levels were obviously increased with high concentration of 0.3 *μ*M C3a for a prolonged 72-hour time. This finding suggested that the response of RPE cells to trigger angiogenesis after complement stimulation requires a specified concentration and incubation time.

After confirming that exposure to a high dose of exogenous C3a for a long time triggers an increased expression of CCN2/CTGF, we next used siRNA to knockdown endogenous C3a in the RPE cells. Compared to nontransfected cells, there was a significant reduction in CCN2/CTGF mRNA after C3-siRNA transfection with the 0.3 *μ*M C3a treatment for 24, 48 and 72 hours. This result suggested that C3 siRNA could reverse the increased transcription of CCN2/CTGF mRNA caused by exogenous C3a stimulation to some extent, and this inhibitory effect may be due to the abolished effect of endogenous C3a by C3 silencing. Moreover, the CCN2/CTGF protein only decreased significantly after siRNA silencing with the 0.3 *μ*M C3a treatment for 48 hours, which was inconsistent with the decreased mRNA levels. This result was probably due to the different regulatory mechanisms of synthesised mRNA and protein (such as synthesis and degradation rates), which means that the relationship between mRNA and protein might not be strictly linear [29].

In accordance with our previous findings, exogenous C3a increased VEGF [28], one of the key mediators of angiogenesis. The upregulation of VEGF and CCN2/CTGF by exogenous C3a confirmed that cells under immune activation were signals for angiogenesis and fibrosis scar formation, which suggests that reduction of C3 could be a potential antiangiogenic treatment [30]. Nevertheless, there are some concerns about C3 as a target for therapy. Complex effects were observed in a phase 2 study using pegcetacoplan, which binds to C3 and consequently controls the cleavage of C3 for geographic atrophy (GA). The patients enrolled showed a slowed GA growth, but the co-occurrence of new-onset retinal exudation resulting from CNV was dose dependent [31], and there were more frequent endophthalmitis-related events [31]. A possible reason is that C3 inhibition decreases the innate immune system response, remitting photoreceptors and RPEs and may also include the underlying choriocapillaris for neovascular growth. Hence, application of C3 inhibition as a potential therapy for angiogenesis and fibrosis scar formation needs further careful investigation [32].

Our study also possessed some limitations. First, the gene silence effect of C3 siRNA was only examined using RT-PCR but should be further validated with western blotting and immune fluorescence. Second, the interactions between C3 and CCN2/CTGF were only investigated in a cell line model but should be further verified in patient-derived samples or animal models of retinal fibrovascular diseases. Finally, the underlying involvement of the signalling pathways related to the regulatory effect of C3 on CCN2/CTGF has not been well elucidated and requires further investigation.

In conclusion, long-term exposure to specified concentration of exogenous C3a increased CCN2/CTGF expression in human RPE *in vitro*, and C3 siRNA could reverse the above elevated CCN2/CTGF mRNA and protein levels to some extent. This result provides important evidence of the association between complement stimulation and CCN2/CTGF and provides C3 inhibition as a novel insight into the therapy strategy for ocular angiogenesis and fibrotic scar formation.

## Figures and Tables

**Table 1:** PCR primers used in this study.

Gene	Forward primer (5′ -3′)	Reverse primer (5′ -3′)
GAPDH	TCACCATCTTCCAGGAGCGAG	TGTCGCTGTTGAAGTCAGAG
Complement 3a	GCTGAAGCACCTCATTGTGA	CTGGGTGTACCCCTTCTTGA
CCN2/CTGF	AAGGACTCTCCGCTGCGGTA	GTGCACCGCCAAAGATGGT

## Data Availability

The datasets obtained and/or analyzed during the current study are available from the corresponding author on reasonable request.
